# Minocycline and sulforaphane inhibited lipopolysaccharide-mediated retinal microglial activation

**Published:** 2007-07-09

**Authors:** Li-ping Yang, Xiu-an Zhu, Mark O.M. Tso

**Affiliations:** 1 Peking University Eye Center, Peking University Third Hospital, Peking University, Beijing, P.R. China; 2 Wilmer Eye Institute, Johns Hopkins University School of Medicine, Baltimore, MD

## Abstract

**Purpose:**

To elucidate the inhibitory effect of minocycline and sulforaphane on lipopolysaccharide (LPS)-induced retinal microglial activation and the mechanisms through which they exerted their inhibitory effects.

**Methods:**

Primary retinal microglial cultures were exposed to LPS with or without minocycline and sulforaphane. The mRNA expression of monocyte chemotactic protein (MCP)-1, MCP-3, macrophage inflammatory protein (MIP)-1α, MIP-1β, eotaxin, regulated upon activation normal T-cell expressed and secreted (RANTES) protein, and interleukin (IL)-10 were examined by reverse transcription polymerase chain reaction (RT-PCR) assay. The mRNA expression of inducible nitric oxide synthase (iNOS) and subsequent nitric oxide (NO) production were examined by RT-PCR assay and Griess reagent assay. Protein expression of the p65 subunit of nuclear factor-κB (NF-κB) and p-p38, p-p44/42 and p-JNK mitogen-activated protein kinases (MAPKs) were examined by Western blot and immunofluorescent analysis.

**Results:**

Cultured retinal microglial cells were activated following exposure to LPS. The mRNA expression and protein production of eotaxin, RANTES, and IL-10 and the mRNA expression of iNOS and subsequent NO production were upregulated. The protein expression of p-p38, p-JNK, and the p65 subunit of NF-κB were also upregulated. However, the protein expression of p-p44/42 was not significantly changed. Pretreatment with minocycline or sulforaphane for 1 h before LPS administration inhibited LPS-induced microglial morphological change and inhibited LPS-induced upregulation of p-p38, but had no effect on the expression of p-p44/42, p-JNK, and the p65 subunit of NF-κB.

**Conclusions:**

Minocycline and sulforaphane inhibited LPS-induced retinal microglial activation, Western blot and immunofluorescent studies showed decreased p-p38 MAPK expression. We suggested that the inhibitory effect of minocycline and sulforaphane was partly through a p38 MAPK-dependent mechanism.

## Introduction

Microglia, major glia of the central nervous system (CNS), play a critical role as resident immunocompetent and phagocytic cells in the CNS [1]. Presence of activated microglia was demonstrated in pathological lesions in several neurological and retinal diseases including Alzheimer's disease, Parkinson's disease [2], multiple sclerosis [3] and retinal degeneration [4,5]. Although microglia promoted neuronal cell viability and survival by producing growth factor and removing potentially toxic cellular debris [6], there was also evidence that activated microglia was deleterious to neurons through excessive production of inflammatory mediators [5,7]. Microglia and their secretions were major contributors to the enhanced death of neurons in neurodegenerative diseases [8]. Hence, understanding the secretion of microglia and the mechanisms regulating microglial activation is an important step in developing therapeutic strategies that ameliorate symptoms of these diseases. Studies demonstrated that brain-derived microglial cells released immuno-regulatory and neuroprotective agents in interaction with phosphatidyl serine-expressing apoptotic cells [9]. However retinal microglial cells promoted photoreceptor death in vitro [10,11]. Previous studies mainly reported the expression of cytokines/chemokines in brain microglia, which was different from retinal microglia. In this study the cultured retinal microglia was used to study microglial activity. Lipopolysaccharide (LPS) is used as a tool to simulate a challenge by gram-negative bacteria and to study the microglial activation process.

Minocycline, a semi-synthetic, long-acting tetracycline derivative with good penetration of the blood-brain barrier, has recently been shown to have remarkable neuroprotective properties in models of neurodegeneration [12,13], brain ischemia [14], and multiple sclerosis [15]. Aside from its direct anti-apoptosis effect, this neuroprotective function was also associated with reduced activation of microglia and reduction of inducible nitric oxide synthase (iNOS) and interleukin-1β (IL-1β)-converting enzyme (ICE) expression [4,14]. However, the mechanism regulating this inhibition was not clear.

Sulforaphane, a naturally occurring cancer chemopreventive agent found in broccoli [16], has been shown to suppress LPS-mediated expression of iNOS, Cox-2 and tumor necrosis factor-α (TNF-α) in Raw 264.7 macrophage cells [17]. In view of this observation, we hypothesized that sulforaphane may modulate the inflammatory response of activated retinal microglia. Because sulforaphane occurs naturally in the widely consumed vegetable broccoli, this might provide a convenient approach to militate retinal degenerative diseases.

In the present study, we investigated (1) the expression of immunological signaling molecules in cultured retinal microglia with or without LPS treatment; (2) the cellular pathways regulating the LPS-mediated microglial activation processes; and (3) the inhibitory effect of minocycline and sulforaphane on LPS-mediated microglial activation and the mechanisms through which they exert their effects.

## Methods

### Primary retinal microglial culture

A primary culture of murine retinal microglial cells was prepared from newborn Sprague-Dawley rat retinas according to the technique by Roque and Caldwell [18], with minor modifications. Briefly, the eyes were enucleated and dissected, the retinas were peeled out and incubated in Ca^++^/Mg^++^-free Hank's balanced salt solution containing 0.25 mg/ml trypsin and 0.4 mg/ml EDTA at 37 °C for 20 min (Sigma-Aldrich, St. Louis, MO). Afterwards, enzyme-treated tissues were dissociated into single cells by gentle pipetting and centrifuged. The dissociated cells were resuspended in DMEM/F-12 (1:1; Invitrogen Corporation, Grand Island, NY, USA) containing 10% fetal bovine serum (FBS) and 1% penicillin/streptomycin (Sigma-Aldrich). Cells were seeded at a density of 10^6^ cells/ml in T75 culture flasks (Corning Incorporation, Corning, NY) and incubated at 37 °C in a humidified atmosphere of 5% CO_2_ and 95% air. After 2-3 weeks in vitro, microglial enriched cultures were shaken at 200 rpm on an orbital shaker (Lab-Line Instruments, Melrose Pk, IL) for 4 h and harvested. Microglial cells were reseeded in poly-L-lysine-coated 35 mm culture dishes (Corning Incorporation, Corning, NY).

### Immunofluorescent studies

For immunofluorescence, the cultures were fixed with 4% paraformaldehyde in 0.01 M phosphate-buffered saline (PBS) for 30 min and rinsed with PBS. The cells were permeabilized with cold (-20 °C) methanol for 10 min. After washing with PBS, they were incubated with blocking buffer (1% bovine serum albumin) for 3 h at room temperature. Subsequently, the cultures were incubated with primary antibodies to microglia (CD11b, Serotec Ltd., Oxford, UK), astrocytes (GFAP, Santa Cruz Biotechnology, Santa Cruz, CA), Müller cells (CRALBP, Santa Cruz Biotechnology), the p65 subunit of NF-κB (Santa Cruz Biotechnology, Santa Cruz, CA), and the p-p38, p-p44/42, and p-JNK mitogen-activated protein kinases (MAPKs; Promega Corporation, Madison, WI) overnight at 4 °C. After rinsing with PBS, the cultures were incubated with the appropriate secondary antibodies for 45 min and examined by fluorescent microscopy.

### Cell exposure experiment

1. Activation of microglia with LPS: Harvested microglial cells were seeded onto 35 mm culture dishes (Corning Incorporation, Corning, NY) and allowed to grow for 2 days. After three washes with PBS, they were incubated with serum-free medium (Invitrogen Corporation, Grand Island, NY) containing 200 ng/ml LPS (Sigma-Aldrich) [19]. The cultures were exposed for 24 h unless otherwise stated.

2. Treatment with pharmaceutical agents: In some experiments, microglial cultures were exposed to minocycline (Sigma-Aldrich, St. Louis, MO), sulforaphane (LKT laboratories, St. Paul, MN), or SB203580 (a specific inhibitor of p38 MAPK, Sigma-Aldrich) for 1 h before administering LPS. To determine a dose-response curve for different concentrations of minocycline on the extent of LPS stimulation, the cultures were treated with 2.5 μM, 0.625 μM, 0.16 μM, 0.04 μM, 0.01 μM, and 0.0025 μM minocycline for 1 h and then exposed to LPS. To determine a dose-response curve for different concentrations of sulforaphane on the extent of LPS stimulation, the cultures were treated with 80 μM, 40 μM, 20 μM, 10 μM, 5 μM, 2.5 μM, 1.25 μM, and 0.32 μM sulforaphane for 1 h and then exposed to LPS. Minocycline was dissolved in PBS and diluted with serum-free medium. Sulforaphane and SB203580 were dissolved in dimethyl sulfoxide (DMSO, Sigma-Aldrich) and diluted with serum-free medium before addition to the microtiter plate well. The final concentrations of DMSO were less than 0.1% (by volume).

### Nitric oxide and cytokine assays

The production of nitric oxide (NO) was quantified by measuring the released NO metabolites (nitrate and nitrite) with Griess reagent (Sigma-Aldrich). The culture media samples were collected and prepared cell-free by centrifugation. The media (100 μl) were incubated with the same volume of Griess reagent at room temperature for 15 min, and were measured at 570 nm in a microplate reader (Bio-Rad, Richmond, CA) with an appropriate standard. The Griess reagent assays were repeated four times.

Monocyte chemotactic protein (MCP)-1, MCP-3, macrophage inflammatory protein (MIP)-1α, MIP-1β, eotaxin, regulated upon activation normal T-cell expressed and secreted (RANTES) protein, and interleukin (IL)-10 samples were prepared similar to NO samples and determined using rat ELISA Kits (R&D Systems, Minneapolis, MN) according to manufacturer's instructions. These experiments were repeated four times.

### Total RNA extraction and semi-quantitative reverse transcription

The microglial cells were exposed to LPS with or without minocycline or sulforaphane for 1 h, 4 h, 12 h, or 24 h, and harvested. For PCR assay, the cells were plated in 6-well plates with 2x10^6^ cells per well. Total RNA was extracted using the Trizol reagent (Invitrogen Corporation) according to the manufacturer's instructions. Reverse transcription (RT) was performed with oligonucleotide primers using Superscript reverse transcriptase (Invitrogen Corporation) according to the manufacturer's protocol and PCR was performed afterwards. The primers and annealing temperatures are shown in Table 1. Each PCR product was separated on 2% agarose gel and analyzed with Quantity One 1-D Analysis software (Bio-Rad). The amount of RT-PCR product was corrected with β-actin expression. Experiments with series diluted template amplification for 30-35 cycles were just in the linear range of detection of the examined genes, so we chose to amplify all examined genes for 35 cycles. The level of expression was compared within the time course for one particular gene. In each series of experiments (between 0 to 24 h), the highest expression was arbitrarily normalized to 100%. The PCR experiments were repeated four times from separate cultures.

**Table 1:** Oligonucleotides used for reverse transcription polymerase chain reaction.

**Target gene**	**Sequences (5'-3')**	**Location**	**Annealing temperature (°C)**
β-Actin	F: CATCCTGCGTCTGGACCT	NT 603-1082	59
	R: TCAGGAGGAGCAATGATCTTG		
MCP-1	F: ACTGGACCAGAACCAAGTGAGA	NT 342-666	61
	R: TGTTGAACCAGGATTCACAGAGA		
MCP-3	F: GGTTGAGGAGGCCATAGCATAC	NT 329-705	61
	R: CACTGATTCTTGCAATGTCCCT		
MIP-1α	F: GAGCTGGAACTAAATGCCTGA	NT 328-653	61
	R: TCACCAAACACAGTGTGAGCA		
MIP-1β	F: CCTTCTGCGATTCAGTGCTGT	NT 47-256	61
	R: CATACTCATTGACCCAGGGCT		
Eotaxin	F: GCACGCTGAAAGCCATAGTCT	NT 204-557	61
	R: CATCTCCTTCCATGCTCTCTCCT		
Rantes	F: CTGCATCCCTCACCGTCATC	NT 17-242	57
	R: CACTTCTTCTCTGGGTTGGCA		
IL-10	F: AGAAGCTGAAGACCCTCTGGA	NT 385-693	59
	R: TGGAGAGAGGTACAAACGAGGT		
iNOS	F: GCTCCATGACTCTCAGCACAG	NT3164-3509	57
	R: CTCAACCTGCTCCTCACTCAAG		

Primers were designed using the Primer Express Program and purchased from AuGCT Biotechnology, Beijing, P. R. China.

### Western blot analysis

The microglial cells were exposed to LPS with or without minocycline or sulforaphane for 1/3 h, 1/2 h, 1 h, or 2 h, and harvested. For Western blot assay, the cells were plated in 6-well plates with 2x10^6^ cells per well. Proteins were extracted using protein lysis buffer (50 mM Tris-CL pH 8.0, 0.02% sodium azide, 1 μg/ml aprotinin, 1% NP-40, 100 μg/ml phenylmethylsulfonyl fluoride [PMSF]) and the final protein concentrations were determined using the BCA protein assay kit (Pierce Biotechnology, Rockford, IL) according to the manufacturer's specifications. Western blot analysis was then performed as previously described [20]. The antibodies to the p65 subunit of NF-κB, to p-p38, p-p44/42 or p-JNK MAPKs (1:1000 dilution in blocking solution) were used for immunodetection. In some cases, an anti-ERK2 MAPK polyclonal antibody was used to assay total (phospho-independent) ERK and served as a control to ensure that equivalent quantities of proteins were used for SDS-PAGE. To evaluate other proteins, the blot was stripped in stripping buffer (62.5 mM Tris-CL pH 6.8, 2% SDS, 100 mM β-mercaptoethanol) for 30 min at 50 °C and re-probed. The western blot experiments were repeated four times from separate cultures.

### Statistical analysis

Data were presented as the mean±SD. Statistical comparisons were made by single-factor ANOVA. p<0.01 was considered significant.

## Results

### Characterization of mouse retinal microglial cells

**Figure 1:**
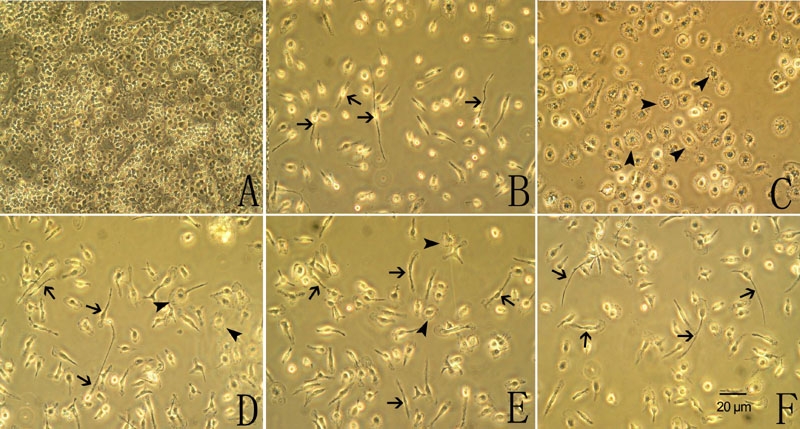
Phase-contrast photomicrographs of the cultured retinal microglial cells. **A**: Mixed cell cultures from newborn Sprague-Dawley rats for 14 days. **B**: Purified cells cultured for 48 h. Cells developed the characteristic ramified shape with a long single process and a small cell soma (arrow). **C**: Purified cells stimulated with LPS for 24 h became bigger and rounder, showing an ameboid shape (arrowhead). Pretreatment with minocycline **D** or sulforaphane **E** for 1 h before LPS administration partly inhibited LPS-induced microglial morphological change, with some cells sustaining their ramified shape (arrow), but a few cells changing to an ameboid shape (arrowhead). **F**: Pretreatment with SB203580 for 1 h before LPS administration completely inhibited the LPS-induced microglial morphological change, with almost all cells sustaining their ramified shape (arrow).

**Figure 2:**
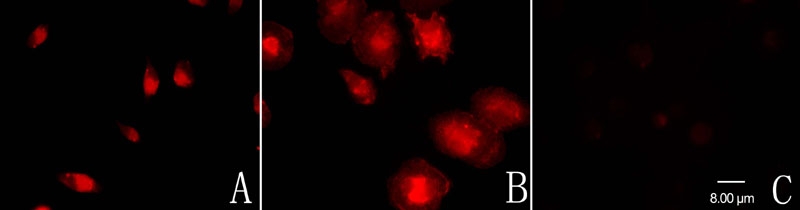
Immunofluorescent characterization of the cultured retinal microglial cells. **A**: The purified cells were reactive to CD11b, a cell-type-specific marker for microglia. **B**: After LPS stimulation for 24 h, the CD11b-labeled cells became bigger and rounder, developing the characteristic ameboid shape of activated microglia. **C**: None of the purified cells showed a positive reaction for GFAP.

The microglial cells were harvested from 14-day-old primary mixed glial cell cultures (Figure 1A) with a "shaking off" method [18]. After purification, cells developed the characteristic ramified shape of resting microglia with a long single process and a small cell soma (Figure 1B). More than 95% of the purified cells showed a positive reaction for CD11b according to immunofluorescent studies (Figure 2A) and none of the cells showed a positive reaction for GFAP (Figure 2C) or CRALBP (data not shown).

### Minocycline and sulforaphane inhibited lipopolysaccharide-induced microglial activation

After LPS stimulation for 24 h, the purified microglial cells became bigger and rounder and developed the characteristic ameboid shape of activated microglia (Figure 1C and Figure 2B). Pretreatment with minocycline (Figure 1D) or sulforaphane (Figure 1E) for 1 h before LPS administration partly inhibited the LPS-induced microglial morphological change, with some cells sustaining their ramified shape, but a few cells showed ameboid shape. Pretreatment with SB203580 for 1 h before LPS administration completely inhibited the LPS-induced microglial morphological change (Figure 1F).

### Minocycline and sulforaphane inhibited mRNA expression of inducible nitric oxide synthase and production of nitric oxide in activated microglial cells

**Figure 3:**
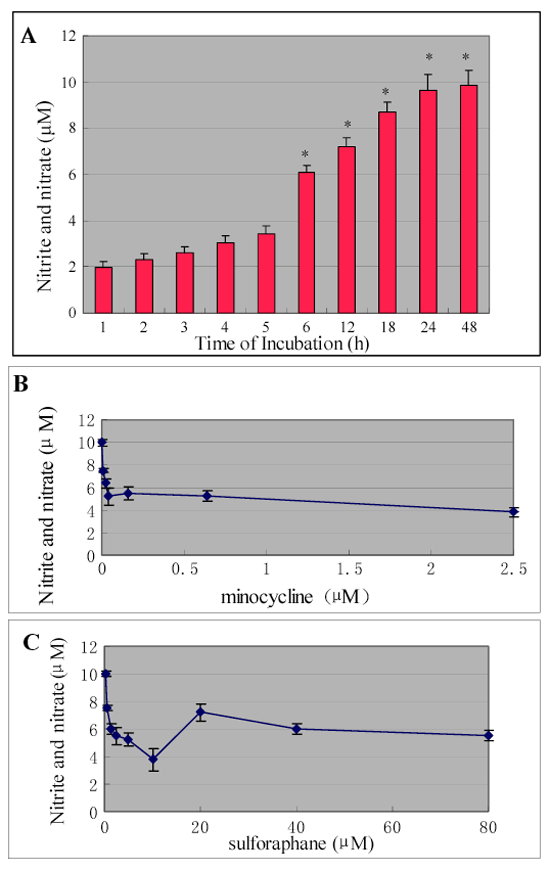
Effect of minocycline and sulforaphane on lipopolysaccharide-induced nitrite and nitrate release in cultured retinal microglial cells. **A**: Release of nitrite and nitrate from cultured retinal microglia with LPS treatment. The increment of nitrite and nitrate levels became significant after 6 h of LPS treatment and continued for up to 24 h. **B**: Administration of minocycline significantly reduced LPS-induced nitrite and nitrate production in retinal microglial cells. **C**: Administration of sulforaphane significantly reduced LPS-induced nitrite and nitrate production in retinal microglial cells. Data represented four independent experiments and were expressed as mean±SD. Asterisk indicates p<0.01 is considered significant.

The effect of minocycline or sulforaphane on LPS-induced iNOS mRNA expression and subsequent NO production in microglial cells was investigated. The microglial cells were treated with LPS alone or with various concentrations of minocycline or sulforaphane. LPS induced a significant time-dependent increment of nitrite and nitrate level in the culture media (Figure 3A). The increment of nitrite and nitrate levels became significant after 6 h of LPS treatment, and continued up to 24 h. Minocycline or sulforaphane itself did not affect the basal iNOS mRNA expression and NO production. The LPS-induced nitrite and nitrate increment was significantly suppressed by administration of minocycline or sulforaphane. The maximal and most consistent inhibition was achieved by treatment with 0.01 μM to 0.625 μM minocycline, or 1.25 μM to 10 μM sulforaphane (Figure 3B,C). The higher doses did not reduce NO production, but had toxic effects on cells. Therefore, 0.16 μM minocycline or 5.0 μM sulforaphane were used for following experiments.

**Figure 4:**
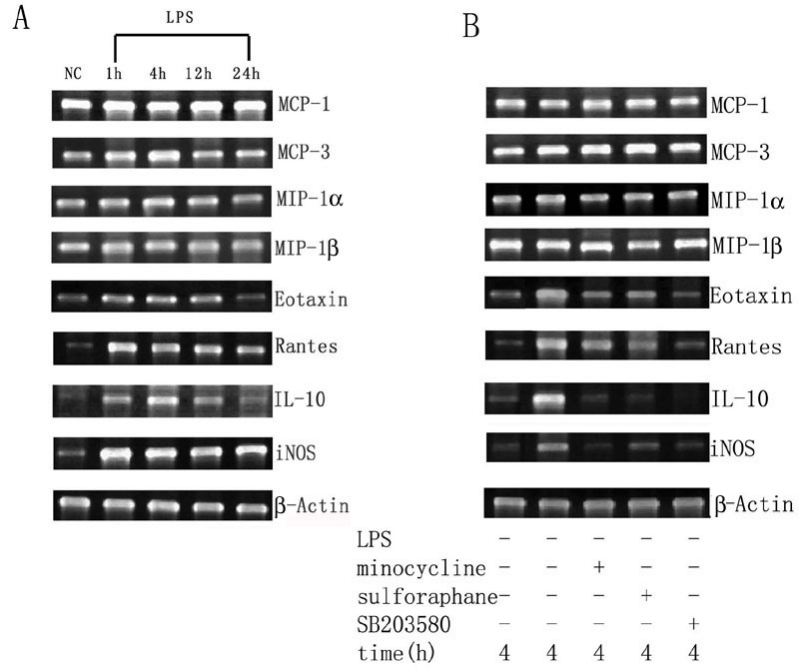
Reverse transcriptase polymerase chain reaction analysis demonstrated the inhibitory effects of minocycline, sulforaphane and SB203580 on lipopolysaccharide-induced mRNA expression of cytokines and inducible nitric oxide synthase in cultured retinal microglial cells. **A**: The mRNA expression of several cytokines and iNOS in cultured retinal microglial cells with or without LPS administration. The retinal microglial cells constitutively expressed modest mRNA transcripts for MCP-1, MCP-3, MIP-1α, and MIP-1β without LPS administration, and treatment with LPS induced the upregulation of eotaxin, RANTES, IL-10 and iNOS. However, the expression levels for MCP-1, MCP-3, MIP-1α, and MIP-1β were little changed. **B**: Pretreatment with minocycline, sulforaphane or SB203580 for 1 h before LPS administration significantly reduced the LPS-induced upregulation of eotaxin, RANTES, IL-10, and iNOS, but had no significant effect on the expression of MCP-1, MCP-3, MIP-1α, and MIP-1β.

To further examine whether minocycline or sulforaphane inhibited NO production via suppression of iNOS mRNA expression, the effect of minocycline or sulforaphane on iNOS mRNA expression in LPS-stimulated microglia was investigated. RT-PCR analysis demonstrated that cultured microglial cells constitutively expressed very low levels of iNOS mRNA without LPS treatment, but expression was upregulated after exposure to LPS (Figure 4A). LPS-induced iNOS mRNA expression was completely suppressed by treatment with minocycline and was mostly suppressed (70%) by treatment with sulforaphane (Figure 4B).

### Minocycline and sulforaphane modulated mRNA expression and protein production of several cytokines in activated microglial cells

The mRNA expression of cytokines in cultured retinal microglial cells with or without LPS treatment was investigated. Retinal microglial cells without LPS administration constitutively expressed modest quantities of mRNA transcripts for MCP-1, MCP-3, MIP-1α and MIP-1β. Following LPS treatment, their expression was slightly upregulated, but the difference was statistically insignificant (Figure 4A). Retinal microglial cells without LPS treatment constitutively expressed low levels of mRNA transcripts for eotaxin, RANTES, and IL-10. Following LPS treatment, their expression was markedly upregulated. The expression of eotaxin and RANTES peaked 1 h after LPS stimulation; however, the expression of IL-10 was much delayed and peaked 4 h after LPS stimulation (Figure 4A).

Minocycline, sulforaphane or SB203580 alone did not affect the basal mRNA transcripts for MCP-1, MCP-3, MIP-1α, MIP-1β, eotaxin, RANTES and IL-10. Pretreatment with minocycline, sulforaphane or SB203580 for 1 h followed by LPS administration significantly reduced LPS-induced upregulation of eotaxin, RANTES, and IL-10, but had no significant effect on the expression of MCP-1, MCP-3, MIP-1α, and MIP-1β (Figure 4B).

The protein production of MCP-1, MCP-3, MIP-1α, MIP-1β, eotaxin, RANTES, and IL-10 in the culture media of microglial cells with or without LPS treatment was examined by ELISA assay. Microglial cells without LPS administration produced modest quantities of MCP-1, MCP-3, MIP-1α and MIP-1β. Following exposure to LPS for 24 h, their production was not changed. Microglial cells without LPS administration produced low levels of eotaxin, RANTES and IL-10. Following exposure to LPS for 24 h, their production was significantly upregulated (Table 2).

**Table 2:** Effects of minocycline, sulforaphane or SB203580 on LPS-induced production of MCP-1, MCP-3, MIP-1α, MIP-1β, eotaxin, RANTES, and IL-10.

**Groups**	**MCP-1**	**MCP-3**	**MIP-1α**	**MIP-1β**	**eotaxin**	**RANTES**	**IL-10**
culture without LPS treatment	180.08±13.21	155.21±8.76	26.34±3.61	43.89±2.78	16.08±4.91	21.44±7.08	15.01±2.46
culture with LPS treatment	192.46±8.67	164.49±14.31	30.17±4.68	40.62±6.34	76.23±9.31	96.39±10.26	70.83±5.69
minocycline pretreated and LPS-exposed culture	204.31±15.29	160.54±9.29	23.64±2.89	49.52±5.73	22.46±5.89*	38.61±5.37*	13.67±2.81*
sulforaphane pretreated and LPS-exposed culture	190.63±8.69	171.38±7.46	24.58±3.74	38.84±4.79	29.36±8.74*	34.46±3.83*	12.34±3.29*
SB203580 pretreated and LPS-exposed culture	176.33±10.21	150.74±8.63	26.01±2.54	36.35±4.81	19.31±4.26*	28.74±3.06*	15.73±4.87*

Asterisk indicates p<0.01 was considered significant compared to the culture with LPS treatment.

Minocycline, sulforaphane or SB203580 alone had no effect on the production of MCP-1, MCP-3, MIP-1α, MIP-1β, eotaxin, RANTES, and IL-10. Pretreatment with minocycline, sulforaphane or SB203580 for 1 h followed by LPS administration significantly decreased LPS-induced production of eotaxin, RANTES and IL-10 in the culture media, but had no significant effect on the production of MCP-1, MCP-3, MIP-1α, and MIP-1β (Table 2).

### The inhibitory effects of minocycline and sulforaphane were associated with the p38 mitogen-activated protein kinases pathway in activated microglial cells

**Figure 5:**
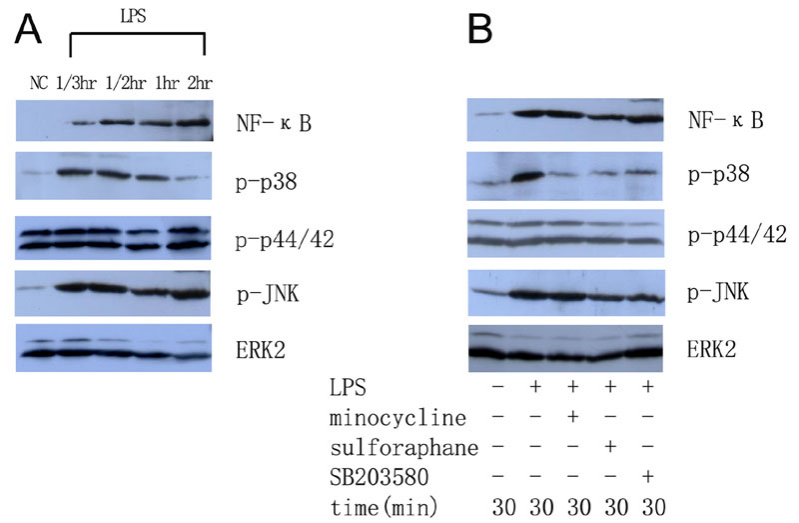
Western blot analysis demonstrated the inhibitory effects of minocycline, sulforaphane and SB203580 on the protein expression of the p65 subunit of nuclear factor-κB and mitogen-activated protein kinases in cultured retinal microglial cells. **A**: The protein expression of the p65 subunit of NF-κB and p-p38, p-p44/42, and p-JNK MAPKs in cultured microglia with or without LPS administration. The cultured retinal microglial cells constitutively expressed high levels of p-p44/42, but expressed low levels of p-p38, p-JNK, and the p65 subunit of NF-κB without LPS administration. Following exposure to LPS, the expression of p-p38, p-JNK, and the p65 subunit of NF-κB was upregulated. However, the expression of p-p44/42 was not changed. The ERK2 MAPK was used to assay total (phospho-independent) ERK and served as a control to ensure that equivalent quantities of proteins were used for SDS-PAGE. **B**: Pretreatment with minocycline, sulforaphane or SB203580 completely inhibited LPS-induced upregulation of p-p38, but had no effect on the expression of p-p44/42, p-JNK, and the p65 subunit of NF-κB.

To examine whether minocycline or sulforaphane inhibit LPS-induced microglial activation via the NF-κB or MAPK pathway, the protein expression of the p65 subunit of NF-κB and the p-p38, p-p44/42, and p-JNK MAPKs in microglial cells with or without minocycline and sulforaphane treatment were investigated. Retinal microglial cells expressed low levels of the p65 subunit of NF-κB without LPS treatment, but expression was upregulated after stimulation with LPS. The p65 subunit was activated within 20 min after LPS stimulation, reached its peak at 30 min, and persisted at this level for least to 2 h. Without LPS treatment, the cells constitutively expressed high levels of p-p44/42, but expressed very low levels of p-p38 and p-JNK. An induced upregulation of p-p38 and p-JNK was demonstrated after stimulation with LPS, but the expression level for p-p44/42 was not changed. The p-p38 MAPK was activated within 20 min after LPS stimulation, started to decline after 1 h, and decreased back to basal levels within 2 h. P-JNK was activated within 20 min after LPS stimulation, reached its peak at 30 min, and persisted at this level for at least to 2 h (Figure 5A).

Pretreatment with minocycline or sulforaphane for 1 h followed by LPS administration completely inhibited the LPS-induced upregulation of p-p38, but had no effect on the expression of p-p44/42, p-JNK, and the p65 subunit of NF-κB (Figure 5B).

### Immunofluorescent studies

**Figure 6:**
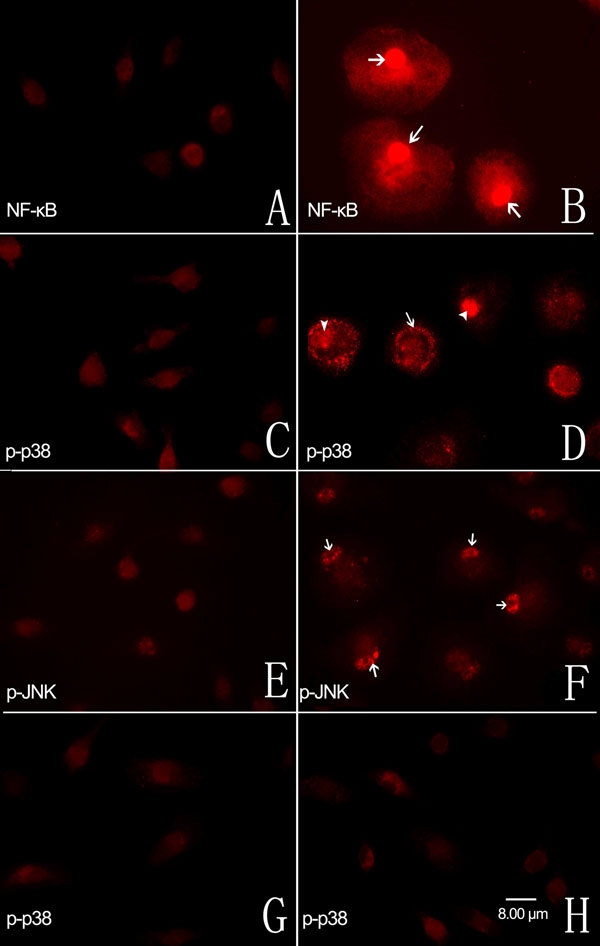
Immunofluorescent localization of the p65 subunit of nuclear factor-κB and the p-p38, and p-JNK mitogen-activated protein kinases in cultured retinal microglial cells. **A**: Cells expressed very low levels of the p65 subunit of NF-κB without LPS administration. **B**: Following exposure to LPS for 1 h, p65 subunit labeling was upregulated and translocated into the nuclei (arrow). **C**: Cells expressed low levels of p-p38 without LPS administration. **D**: Following exposure to LPS for 1 h, p-p38 expression was upregulated and positive labeing was present in both cytoplasm (arrow) and nuclei (arrowhead). **E**: Retinal microglial cells expressed low levels of p-JNK without LPS administration. **F**: Following exposure to LPS for 1 h, the positive labeling of p-JNK was present mainly in nuclei (arrowhead). Pretreatment with minocycline **G** or sulforaphane **H** for 1 h followed by LPS administration completely inhibited LPS-induced upregulation of p-p38. All panels have the same scale and the scale bar equal to 8 μm.

To further confirm the upregulation of p-p38, p-JNK, and the p65 subunit of NF-κB in LPS-stimulated microglial cells, protein levels were studied with immunofluorescence. Retinal microglial cells expressed very low levels of the p65 subunit of NF-κB without LPS administration (Figure 6A). Following LPS administration for 1 h, p65 subunit labeling was upregulated and translocated into nuclei (Figure 6B). Similarly, cells expressed low levels of p-p38 without LPS administration (Figure 6C). Following LPS administration for 1 h, p-p38 labeling was upregulated and positive labeling was present in both cytoplasm and nuclei (Figure 6D). Cells expressed low levels of p-JNK without LPS administration (Figure 6E), but labeling was upregulated following LPS administration for 1 h and was present mainly in the nuclei (Figure 6F). Pretreatment with minocycline (Figure 6G) or sulforaphane (Figure 6H) for 1 h followed by LPS administration completely inhibited LPS-induced upregulation of p-p38, but had no effect on the expression of p-JNK and the p65 subunit of NF-κB (data not shown).

## Discussion

Our study demonstrated (1) the expression of 8 immunological signaling molecules (chemokines and noxious factors) in cultured retinal microglial cells treated with or without LPS; (2) that NF-κB and MAPKs were two cellular pathways involved in LPS-mediated microglial activation processes; and (3) that minocycline and sulforaphane had efficient inhibitory effects on LPS-mediated microglial activation processes, partly through a p38 MAPK-dependent mechanism.

In the present study, the cultured retinal microglial cells changed their morphological shape following LPS administration (Figure 1B,C). The mRNA expression of iNOS and subsequent production of NO were upregulated (Figure 4A and Figure 3A), as was mRNA expression of pro-inflammatory cytokines including eotaxin, RANTES and IL-10. However, mRNA expression of the chemokines MCP-1, MCP-3, MIP-1α, and MIP-1β was not significantly changed (Figure 4A).

Microglial activation and the associated induction of NO and pro-inflammatory cytokines have been well documented in various neurodegenerative diseases, such as Alzheimer's disease and Parkinson's disease [21,22]. NO seemed to play an important role in the pathogenesis of endotoxin-induced uveitis [23] and light-induced retinal degeneration [24]. In the present study, we observed that iNOS mRNA expression and subsequent NO production were upregulated in retinal microglia after LPS treatment, suggested that NO may be an important mediator of the neurotoxic activity exerted by activated retinal microglial cells.

Chemokines and cytokines are immune signaling molecules that may inflict secondary damage during inflammation and exaggerate neurodegeneration [25,26]. Resting microglia are dominant immune surveillant cells which need appropriate stimulation to be transformed into full-blown macrophages [1,27]. The activation process included the synthesis of cytotoxic substances such as oxygen radicals, nitric oxide, proteases, and proinflammatory cytokines [28,29]. Eotaxin and RANTES are members of the C-C chemokine family, which were involved in macrophage recruitment during inflammation [30]. Therefore, the upregulated expression of eotaxin and RANTES in retinal microglia following LPS treatment might be important for the propagation and maintenance of the retinal immune response or for recruitment of more microglia to the lesion. Previous in vivo studies from Zeng et al. [5] demonstrated that normal retinas containing resting microglia do not expressed MCP-1, MCP-3, MIP-1α, and MIP-1β. However, their expression was upregulated in retinal degeneration. In the present study, we demonstrated that the cultured retinal microglial cells without LPS treatment constitutively expressed modest quantities of MCP-1, MCP-3, MIP-1α, and MIP-1β, and their expression was little changed after LPS treatment. This might be because cultured microglial cells in vitro exhibit a reactive phenotype and produce cytokines that are specific for reactive microglia [31]. The constitutively expressed chemokines in these cells might be due to the preparation procedure for the culture. Further in vivo studies are needed to illustrate their role in retinal disease.

It is accepted that the production of pro-inflammatory cytokines in the CNS is under strict control to maintain normal homeostasis of the CNS. We demonstrated that retinal microglial cells also secreted the anti-inflammatory cytokine IL-10 after LPS treatment, which suppressed the activated microglia. The above observation strongly supported that retinal microglial cells play an important role in the propagation and maintenance of the retinal immune response. The upregulation of several chemokines in retinal microglial cells following treatment with LPS suggested the involvement of complicated immunological mechanisms in the retinal disease process.

In this study, LPS treatment upregulated the expression of the p65 subunit of NF-κB and the p-p38 and p-JNK MAPKs, but did not change the high levels of p-p44/42 MAPK expression (Figure 5 and Figure 6). NF-κB is a ubiquitous transcription factor that regulates a broad range of genes and is a central regulator of microglial response to activating stimuli, including LPS and cytokines [32]. One previous study demonstrated that, upon stimulation, IκB phosphorylation and degradation allowed NF-κB to translocate into the nucleus and recognize consensus sequences in target genes, including iNOS, IL-1β, and TNF-α [33]. Consistent with this finding, our observation demonstrated that the NF-κB pathway played a role in the LPS-induced retinal microglial activation process.

MAPKs are serine/threonine kinases and form one of the major pathways linking extracellular stimuli to transcriptional activation and gene expression. The p-p38 MAPK pathway is involved in the induction of several pro-inflammatory genes and p-p38 and p-JNK are always activated simultaneously [34]. In this study, we demonstrated that LPS stimulation induced the upregulation of p-p38 and p-JNK in retinal microglial cells, and p-p38 and p-JNK were activated simultaneously. SB203580, a specific inhibitor of p-p38, inhibited the LPS-induced retinal microglial morphological change and modulated cytokine expression. We suggested that the activation of p-p38 in retinal microglia by LPS is the first step in initiating the inflammatory process, resulting in the production of superoxide radicals and cytokines, which further stimulate the inflammatory response. The studies of Fukuda et al. [35] and Robinson et al. [36] showed that a constitutively active and nuclear form of p44/42 was sufficient for neurite outgrowth and cell transformation, and up to 80% of prostate cells in the stroma and basal layers stained positively for p-p44/42 within the nucleus [37]. In contrast, Combs et al. [38] argued that p-p44/42 might play a critical role in harmful microglial activation in acute brain injury, such as stroke, and in more chronic neurodegenerative disease, such as Alzheimer's disease. Our observations demonstrated that p-p44/42 was constitutively expressed in cultured retinal microglial cells without LPS administration. LPS administration did not alter its activity, suggesting that p44/42 does not play an important role in microglial activation in retinal disease.

Studies by Tang et al. [39] and Liu et al. [40] showed that the MAPK and NF-κB pathways, which were almost invariably co-activated by cytokines and stress, were intimately linked. One of the major molecules for p65 phosphorylation and transactivation appeared to be p-p38 [41]. Saccani et al. [42] also suggested that p-p38 further enhances NF-κB signaling via direct modification of histone proteins in the cryptic κB-regulatory element. In the present study, we demonstrated that SB203580 had no effect on the expression of p-p44/42, p-JNK, and the p65 subunit of NF-κB. It might be that the cellular pathways of NF-κB and MAPKs are relatively separate in LPS-induced microglial activation.

Minocycline was an anti-inflammatory agent which was used in the management of inflammatory and degenerative disease. It was shown to inhibit retinal microglial activation and migration in photic injury. Being a second-generation, semi-synthetic tetracycline with broad-spectrum antimicrobial activity, minocycline was shown to exert biological effects completely distinctive from tetracycline [43]. The mechanism by which minocycline suppressed LPS-induced activation of microglia is not clearly understood, but might be relate to the anti-inflammatory effect and inhibitory effect on microglial activation and migration. In contrast, sulforaphane inhibited phase enzymes such as cytochrome P450 and induces phase detoxification enzymes [44-46]. Intraperitoneal pretreatment with sulforaphane was found to attenuate light-induced retinal damage via an antioxidant-responsive element/thioredoxin signaling cascade [47]. It also suppressed inflammatory reaction in macrophage [17]. In this study, pre-treatment with minocycline or sulforaphane followed by LPS administration inhibited LPS-induced microglial morphological change and decreased mRNA expression of iNOS and subsequent NO production. They also inhibited LPS-induced upregulation of p-p38, but had no significant effect on the expression of p-p44/42, p-JNK, and the p65 subunit of NF-κB. Banati et al. [29] proposed that activated microglia exert cytotoxic effects in the brain through two very different yet complementary processes. First, they act as phagocytic cells, which involves direct cell-to-cell contact. Second, they are capable of releasing a large variety of potentially harmful substances. Our study suggested that the inhibitory effect of minocycline and sulforaphane on LPS-induced microglial activation suppressed the phagocytic activity and decreased the expression of inflammatory mediators. This inhibitory effect was associated with a p38-dependent pathway.

In conclusion, our study demonstrated that minocycline and sulforaphane inhibited LPS-induced retinal microglial activation, western blot and immunofluorescent studies showed decreased p-p38 MAPK expression. We suggested that the inhibitory effect of minocycline and sulforaphane was partly through a p38 MAPK-dependent mechanism. Given the fact that microglial activation contributes to the pathogenesis of several retinal disorders, minocycline or sulforaphane may be potential therapeutic agents for inflammatory or degenerative retinal diseases.
